# Prehospital monitoring of cerebral circulation during out of hospital cardiac arrest ? A feasibility study

**DOI:** 10.1186/s13049-022-01044-y

**Published:** 2022-12-02

**Authors:** Anna Henningsson, Lukas Lannemyr, Oskar Angerås, Joakim Björås, Niklas Bergh, Johan Herlitz, Bengt Redfors, Peter Lundgren

**Affiliations:** 1 Region Västra Götaland, Sahlgrenska University Hospital, Section of Cardiothoracic Anaesthesia and Intensive Care, Göteborg, Sweden; 2 Department of Anesthesiology and Intensive Care Medicine, Sahlgrenska Academy, University of Gothenburg, Gothenburg, Sweden; 3 Department of Molecular and Clinical Medicine, Institute of Medicine, Sahlgrenska Academy, University of Gothenburg, Gothenburg, Sweden; 4 Region Västra Götaland, Sahlgrenska University Hospital, Department of Cardiology, Gothenburg, Sweden; 5 Prehospen - Centre for Prehospital Research, University of Borås, Borås, Sweden

**Keywords:** Cerebral oximetry, Regional cerebral oxygen saturation, Cardiac arrest, EMS

## Abstract

**Background:**

About two-thirds of the in-hospital deaths after out-of-hospital cardiac arrests (OHCA) are a consequence of anoxic brain injuries, which are due to hypoperfusion of the brain during the cardiac arrests. Being able to monitor cerebral perfusion during cardiopulmonary resuscitation (CPR) is desirable to evaluate the effectiveness of the CPR and to guide further decision making and prognostication.

**Methods:**

Two different devices were used to measure regional cerebral oxygen saturation (rSO2): INVOS™ 5100 (Medtronic, Minneapolis, MN, USA) and Root® O3 (Masimo Corporation, Irvine, CA, USA). At the scene of the OHCA, advanced life support (ALS) was immediately initiated by the Emergency Medical Services (EMS) personnel. Sensors for measuring rSO2 were applied at the scene or during transportation to the hospital. rSO2 values were documented manually together with ETCO2 (end tidal carbon dioxide) on a worksheet specially designed for this study. The study worksheet also included a questionnaire for the EMS personnel with one statement on usability regarding potential interference with ALS.

**Results:**

Twenty-seven patients were included in the statistical analyses. In the INVOS™5100 group (n = 13), the mean rSO2 was 54% (95% CI 40.3–67.7) for patients achieving a return of spontaneous circulation (ROSC) and 28% (95% CI 12.3–43.7) for patients not achieving ROSC (p = 0.04). In the Root® O3 group (n = 14), the mean rSO2 was 50% (95% CI 46.5–53.5) and 41% (95% CI 36.3–45.7) (p = 0.02) for ROSC and no ROSC, respectively. ETCO2 values were not statistically different between the groups. The EMS personnel graded the statement of interference with ALS to a median of 2 (IQR 1–6) on a 10-point Numerical Rating Scale.

**Conclusion:**

Our results suggest that both INVOS™5100 and ROOT® O3 can distinguish between ROSC and no ROSC in OHCA, and both could be used in the pre-hospital setting and during transport with minimal interference with ALS.

**Supplementary Information:**

The online version contains supplementary material available at 10.1186/s13049-022-01044-y.

## Introduction

About two-thirds of the in-hospital deaths after cardiac arrest are a consequence of anoxic brain injuries, which are due to hypoperfusion of the brain during the cardiac arrests [1, 2]. Therefore, being able to monitor cerebral perfusion during cardiopulmonary resuscitation (CPR) and advanced life support (ALS) could help to evaluate the effect of resuscitation and to guide prognosis of neurological outcomes. Today, end tidal carbon dioxide (ETCO2) is the best studied surrogate for cardiac output and central circulation during out of hospital cardiac arrest (OHCA). ETCO2 is affected by ventilation rates and tidal volumes but also by the cardiac compression site and quality of the compressions during CPR. ETCO2 during ALS can confirm the correct placement of the endotracheal tube and may indicate a return of spontaneous circulation (ROSC) when ETCO2 increases during CPR [3, 4]. ETCO2 is routinely measured both in-hospital and pre-hospital.

Cerebral oximetry is a non-invasive technique using near infrared spectroscopy to assess regional cerebral oxygen saturation (rSO2) as a surrogate for cerebral circulation. Cerebral oximetry is frequently used during cardiac and vascular surgery to monitor the oxygen supply to the brain during different states of circulation. RSO2 is a surrogate for regional blood flow in the brain and changes in oxygenation is seen promptly, meaning rSO2 also indirectly reflects the central circulation and cardiac output. Cerebral oximetry is not used routinely during cardiac arrest, and its measurement algorithms are not validated on no- or low-flow circulation during cardiac arrests.

Studies of cerebral oximetry in cardiac arrest patients have evaluated the cerebral circulation both for optimization of CPR and for prediction of the outcome. Thus, this method has the potential of providing an indication whether the cerebral circulation is good enough to avoid anoxic brain injury or not during CPR. Evaluating the central and cerebral circulation with cerebral oximetry in the pre-hospital setting could improve patient outcomes. At the same time, it could draw attention away from other important measures during ALS. The feasibility of cerebral oximetry in cardiac arrests has been investigated both in-hospital and pre-hospital for everyday clinical use both in studies with and without extra study personnel dedicated to rSO2 measurements [5–10]. Meta-analyses aiming to find thresholds for predicting ROSC and thresholds for not being able to reach ROSC have concluded that both initial and mean rSO2 were higher in patients who achieved ROSC [11–13]. To our knowledge, no study has evaluated Root® O3 regional oximetry during OHCA. The available data consist of studies with different devices measuring rSO2 from multiple manufacturers with INVOS™ and Equanox™ being the most common. However, each manufacturer uses different methodologies, algorithms, and sensors to analyze the oxyhemoglobin saturation in the tissue. Therefore, it is uncertain if there is a discrepancy between measurements of different oximeter brands. If there are discrepancies, specific recommendations might be needed for each brand of oximeter. We decided to conduct a pre-hospital study on OHCA patients using both INVOS™ 5100 and Root® O3 at the scene of the cardiac arrest and during transportation to hospital, to assess feasibility and usability during this time critical medical emergency.

## Methods

### Study design, settings, and subjects

The study was designed as a prospective observational study. Ethical approval was obtained from the Regional Ethical Review Board in Gothenburg, Sweden (DNR 1145-17). The primary objective was to assess the feasibility of measuring rSO2 in 30 patients using INVOS™ and Root®O3 in addition to ETCO2 in OHCA in an everyday clinical pre-hospital setting. The secondary objective was to run correlational analyses between rSO2 and ETCO2 to ROSC in the pre-hospital phase for INVOS™ and Root®O3, respectively.

The study was conducted with the Emergency Medical Service (EMS) at Sahlgrenska University Hospital in Gothenburg from October 1, 2018 to September 1, 2019. In this EMS, a rapid response car (RRC) is staffed with an anesthesiologist trained in pre-hospital critical care and a specialist nurse, operates Monday to Friday between 7 a.m. to 9 p.m. in addition to the ambulances. The RRC covers an area with approximately 738,000 inhabitants and receives an average of 14 calls per day. The RRC can be dispatched directly from the emergency call center or by a telephone call from an ambulance in need of extra personnel or specialty. When responding to an OHCA, the RRC is always operating with one or two ambulances.

The study inclusion criteria were individuals > 18 years old who had a cardiac arrest during the time the RRC operates. Traumatic cardiac arrests were excluded.

### Equipment/Education

Two different devices were used to measure rSO2: INVOS™ 5100 (Medtronic, Minneapolis, MN, USA) and Root® O3 (Masimo Corporation, Irvine, CA, USA). All EMS personnel working in the RRC received training on how to use both devices prior to patient inclusion. The INVOS™ 5100 measures rSO2 in two wavelengths with 75% representing venous blood and 25% arterial blood. It weighs 4.95 kg, and its battery capacity is 20 min with a 24-hour recharge for the battery. The INVOS™ 5100 measures rSO2 values between 15 and 95%. The Root® O3 measures rSO2 in 4 wavelengths with 70% representing venous blood and 30% arterial blood. The Root® O3 weighs 3.63 kg and has a battery capacity of 4 h with a maximum charge time of 4 h. It measures rSO2 values from 0 to 100%. All studied patients had mechanical compressions performed by LUCAS (Lund University Cardiopulmonary Assist System, Stryker, Lund, Sweden). ETCO2 was measured with the EMMA® capnograph (Masimo Corporation, Irvine, CA, USA) that was connected to an endotracheal tube. All patients were manually ventilated.

### Protocol

At the scene of the cardiac arrest, ALS was immediately initiated by the EMS personnel. The physician or the specialist nurse attached two sensors to the patient’s forehead to measure rSO2. The sensors for cerebral oximetry were attached when it was appropriate during resuscitation either at the scene or during transport to the hospital. The ETCO2 measurement was commenced directly after endotracheal intubation and was manually documented on the worksheet at the same time as the rSO2 values were documented. Measurements continued until ROSC, arrival at the emergency department, or until ALS was terminated at the scene. The INVOS™ 5100 and Root® O3 were used on alternate weeks. Data were also collected from hospital (Melior) and EMS records (Ambulink).

### Data collection

ETCO2 together with rSO2 values were documented manually on a specific worksheet that was created for this study. RSO2 was measured as soon as possible without affecting ALS, meaning it could have been done at the scene of the arrest or during transportation to the hospital. The worksheet included a timeline where all events were registered (LUCAS, defibrillation, intubation, and doses of epinephrine and amiodarone). Initial and changes in heart rhythm, bystander CPR, no-flow time, pupil size, and signs of life/ROSC were documented. If a focused ultrasound examination were performed, the result was documented in the worksheet. Time from call for EMS to EMS arrival and time from call for EMS to hospital arrival were then cross-checked with the EMS journal. The study worksheet also included a question for the RRC personnel with one statement on usability in regard to interference with ALS. The statement was “Applying the sensors for cerebral oximetry took away focus or interfered in a negative way during ALS.” The RRC personnel rated the statement on a 10-point Numerical rating scale with whole number intervals (0 being ”I do not agree’’ and 10 being ”I absolutely agree’’).

### Data analysis

Patient characteristics were described using means and confidence intervals or medians and interquartile ranges, as appropriate. Mean values of rSO2 between the right sensor and left sensor were calculated. Variables were tested for normal distribution with the Kolmogorov-Smirnov test. When the variables were normally distributed, independent samples *t*-tests were used, and when the variables were not normally distributed, the Mann-Whitney U test was used. P-values < 0.05 were considered statistically significant. Due to the exploratory nature of the study, we did not correct for multiple analyses. All analyses were done in SPSS Statistics 26 (IBM, Armonk, NY, USA).

## Results

In total, 33 patients were included in the study. Six patients were excluded because they had ROSC before measurements were registered, leaving 27 patients for the statistical analysis. Four patients were unidentified, or their social security number was not registered in the pre-hospital chart, making it impossible to collect demographic variables and outcomes at follow up. We managed to measure rSO2 in all but three patients. RSO2 values in these three patients could not be measured due to low battery capacity and/or connection problems between the sensors and monitor. In 24 patients, we had 1–12 measurements per patient with a median number of measurements at three per patient (IQR 2–4). A mean rSO2 was calculated for each patient. Overall, there were no statistically significant differences between the INVOS™5100 and Root® O3 groups in patient characteristics, except for ETCO2, which was significantly higher in the INVOS™5100 group (Table 1).

**Table 1:** Patient’s characteristics

	INVOS n = 13	Root O3 n = 14	P
Age, (mean ± CI)*	65.2 (54-76.4)	70.2 (56.7–83.7)	0.58
Male, n (%)*	7 (58.3)	9 (81.8)	0.37
Bystander CPR n (%)	10 (76.9)	12 (85.7)	0.65
No flow time, min (mean ± CI)	1.1 (0.7–1.5)	2.0 (0.5–3.5)	0.32
Shockable rhythm, n (%)	4 (30.8)	7 (50)	0.44
Time to ambulance, min (mean ± CI)	10.5 (8.1–12.9)	11.0 (8.7–13.3)	0.79
Time to hospital, min (mean ± CI)	46.9 (39.8–54)	48.5 (41.5–55.5)	0.76
Adrenalin, mg (mean ± CI)	4.1 (2.7–5.5)	4.9 (3.4–6.4)	0.46
Dilatated pupils, n (%)	7 (53.8)	11 (84.6)	0.20
rSO2, % (mean ± CI)**	40 (27.2–52.8)	45 (41.3–48.7)	0.42
ETCO2, kPa (mean ± CI)***	4.9 (3.7–6.1)	3.0 (2.6–3.4)	0.01
ROSC, n (%)	7 (58.3)	7 (50.0)	1.00
30 days survival, n (%)****	1 (8)	1 (7)	1.00

*Number of patients in INVOS group were 12 and in ROOT O3 group 11. **Number of patients in INVOS group 11, ROOT O3 group 13. *** Number of patients in INVOS group 11, in ROOT O3 group 10 patients. ****Number of patients in INVOS group 12, in ROOT O3 group 12

In the INVOS™5100 group (n = 13), the mean rSO2 was 54% (95% CI 40.3–67.7) for patients achieving ROSC and 28% (95% CI 12.3–43.7) for patients not achieving ROSC (p = 0.04). In the Root® O3 group (n = 14) mean rSO2 was 50% (95% CI 46.5–53.5) and 41% (95% CI 36.3–45.7) (p = 0.02) for ROSC and no ROSC, respectively. (Table 2).

**Table 2:** Patients’ characteristics in subgroups

	INVOS n = 13			Root O3 n = 14		
	ROSC n = 7	nROSC n = 6	P	ROSC n = 7	nROSC n = 7	P
Age, (mean ± CI*)	69.6 (53-86.2)	60.5 (52.7–68.3)	0.44	62 (38.7–85.3)	77 (62.2–91.8)	0.33
Male, n( %*)	4 (66.7)	3 (50)	1.00	5 (100)	4 (66.7)	0.46
Bystander CPR, n( %)	4 (57.1)	6 (100)	0.19	6 (85.7)	6 (85.7)	1.00
No flow time, min (mean ± CI)	0.8 (0.4–1.2)	1.3 (0.7–1.9)	0.20	1.2 (0,3-2.1)	2.8 (0-5.7)	0.36
Shockable rhythm, n (%)	2 (28.6)	2 (33.3)	1.00	5 (71.4)	2 (28.6)	0.29
Time to ambulance, min (mean ± CI)	12.8 (9.9–15.7)	7.8 (5-10.6)	0.03	10.6 (8-13.2)	11.4 (7.3–15.5)	0.73
Time to hospital, min (mean ± CI)	43.3 (32-54.6)	50.0 (42.5–57.5)	0.29	47.3 (35.2–59.4)	50.2 (43.2–57.2)	0.71
Adrenalin, mg (mean ± CI)	2.7 (1.2–4.2)	5.5 (3.5–7.5)	0.06	5 (3–7)	4.9 (2.6–7.2)	0.93
Dilatated pupils, n (%)	3 (42.9)	4 (66.7)	0.59	5 (71.4)	6 (100)	0.46
rSO2, %(mean ± CI**)	54 (40.3–67.7)	28 (12.3–43.7)	0.04	50 (46.5–53.5)	41 (36.3–45.7)	0.02
ETCO2, kPa (mean ± CI***)	5.1 (3.3–6.9)	4.6 (2.8–6.4)	0.68	3.3 (2.8–3.8)	2.6 (1.9–3.3)	0.19

*Number of patients in the INVOS group 12 and in ROOT O3 group 11. **Number of patients in INVOS group 11, ROOT O3 group 13. *** Number of patients in INVOS group 11, in ROOT O3 group 10 patients

**Fig. 1:**
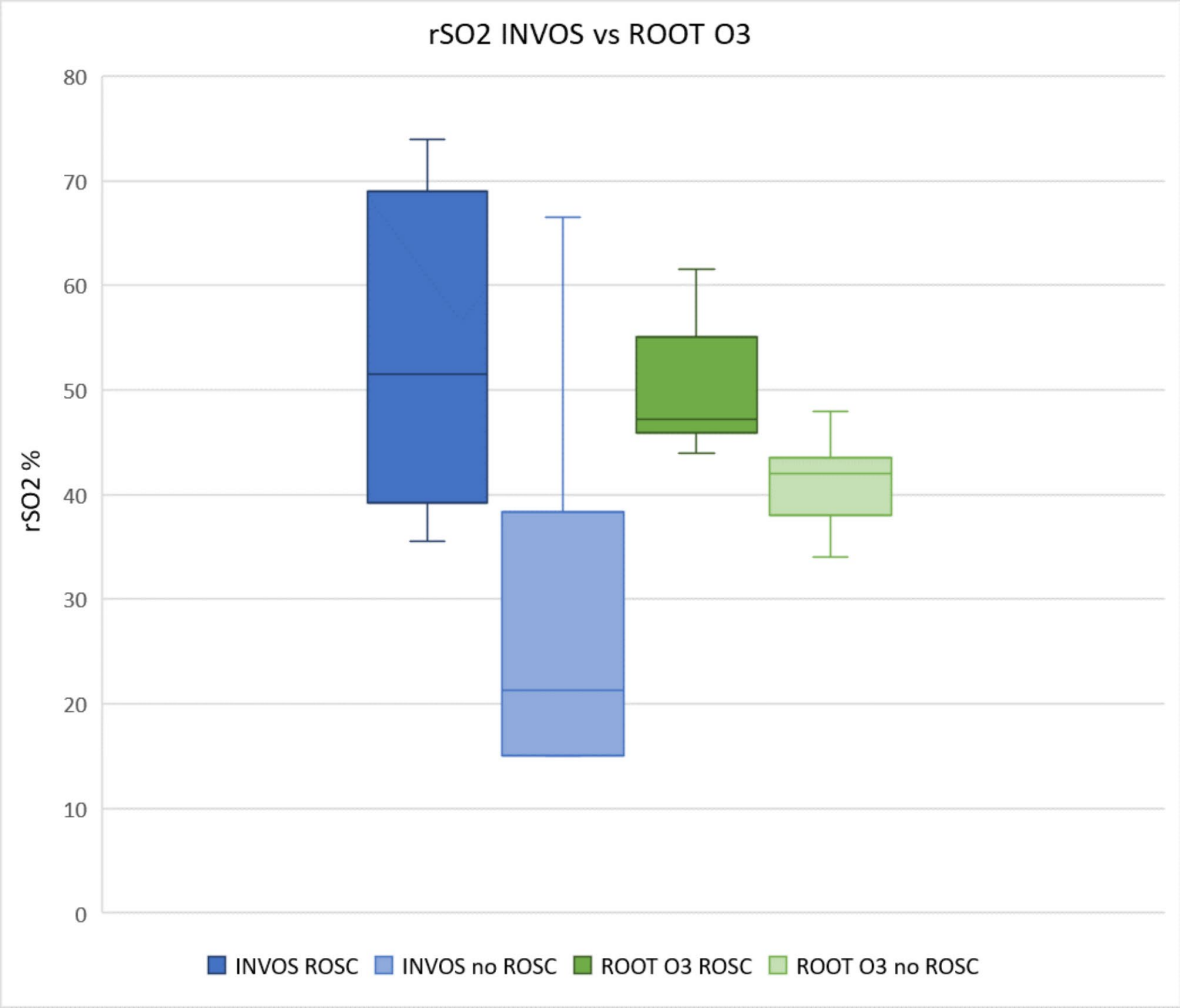
Mean rSO2 values for all patients for INVOS™5100 and ROOT®O3.

The INVOS™5100 group patients achieving ROSC had a mean rSO2 between 36 and 74% and patients with no ROSC 15–67%. Except for one outlier, all patients in the INVOS™5100 group who did not achieve ROSC had a mean rSO2 lower than all patients in the ROSC group. In the ROOT®O3 group, patients who achieved ROSC had a mean rSO2 between of 44–62% and those with no ROSC had a mean rSO2 between 34 and 48%. Except for one patient, all patients in the ROOT®O3 group not achieving ROSC had lower rSO2 values than all patients in the ROSC group. (Fig. 1).

Mean ETCO2 was not statistically significant different between the groups. In the INVOS 5100™ group, the mean ETCO2 when ROSC was achieved were 5.1 kPa and 4.6 kPa when ROSC was not achieved (p = 0.68). In ROOT O3®, the mean ETCO2 was 3.3 kPa in the ROSC group and 2.6 kPa when ROSC was not achieved (p = 0.19). (Table 2).

**Fig. 2:**
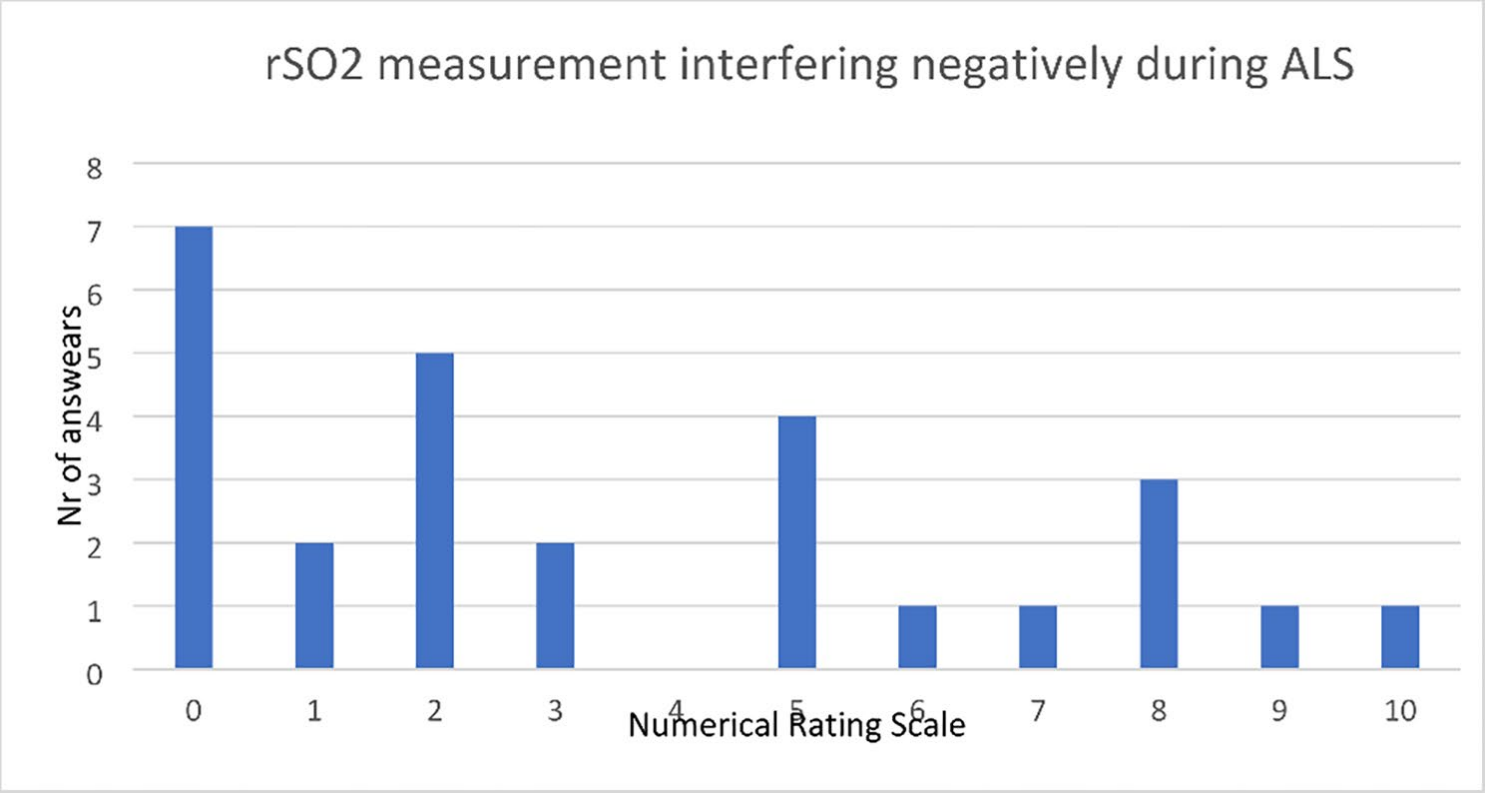
RRC personnel answers to the statement “I experienced applying the sensors for cerebral oximetry to take focus and interfere in a negative way during ALS”.

From the question for the RRC personnel on usability, there were 27 answers to the question about interference with ALS rated according to the Numerical Rating Scale from. 0–10. The median answer was 2 (IQR 1–6) indicating there was very little interference with ALS according to the personnel taking the measurements. (Fig. 2).

## Discussion

Our study showed a statistically significant difference in mean rSO2 between OHCA patients who achieved ROSC and not, using both the INVOS™ 5100 and ROOT® O3. We did not find a difference in ETCO2 between the groups achieving ROSC or not. Both the INVOS™ 5100 and ROOT® O3 can be used in a pre-hospital setting and during transport with minimal interference of ALS according to EMS personnel.

The INVOS™5100, ROOT® O3 and other cerebral oximeters on the market have their own algorithms, methodologies, and sensor designs for analyzing oxyhemoglobin. They also have different ratios for approximation of the contributed arterial and venous saturation (INVOS™ 25/75% and ROOT O3® 30/70%). To the best of our knowledge, all cerebral oximeters are validated during a state of normal circulation. During a cardiac arrest, there is hypocirculation, and the ratio of venous and arterial blood in the cerebral circulation might be different than the ratio 25/75% and 30/70%. When using a device that is not validated for a specific situation, such as resuscitation, this need to be taken into consideration.

We found a statistically significant difference between ROSC and no ROSC with both cerebral oximeters. When rSO2 values were placed in a boxplot, there appears to be a cutoff in rSO2 between 30 and 35% measured with INVOS™5100 compared to the no ROSC group where all patients, except one outlier, had an rSO2 < 30%. All patients achieving ROSC had an rSO2 > 35%. A similar pattern was seen in the Root® O3 group but with a smaller range in rSO2. In the ROOT®O3 group, patients with no ROSC had an rSO2 < 48% and in the ROSC group rSO2 > 44%, and only one patient with no ROSC had an rSO2 > 44%. The lowest mean rSO2 in the ROOT®O3 group was 34% compared with 15% in the INVOS™ 5100 group. As pictured in Fig. 1, a cutoff between ROSC and no ROSC seems to be different for the two devices.

The difference between different cerebral oximeters has been shown in hypocapnia and normal circulation. Bickler et al. compared five different cerebral oximeters with SaO2 and SvO2 and showed a between-subject and between-instrument difference in normocapnic hypoxia [14]. It has also been found that Fore-Sight (CAS Medical Systems, Branford, CT, USA,), Equanox (Nonin Medical, Inc., Plymouth, MN, USA) and NIRO (Hamamatsu Photonics, Hamamatsu-shi Shizuoka, Japan) give an overestimation of rSO2 when compared to calculated values from SaO2 (oxygen saturation) and SvO2 (mixed venous oxygen saturation). Ferraris et al. showed that the rSO2 measured on the forearm during cardiac surgery was not interchangeable between oximeters using Equanox™ and ROOT® O3 [15]. Both INVOS™ and ROOT® O3 are validated during normocapnic hypoxia. Schober et al. showed increasing bias in rSO2 when hypocapnia occurs together with hypoxia measured by Fore-Sight and INVOS [16].

Our results are in line with previous studies showing a statistically significant difference in rSO2 between patients achieving ROSC or not after OHCA [5–9, 17–21]. To the best of our knowledge, only three other studies have incorporated cerebral oximetry in everyday clinical practice in a pre-hospital setting, but they used INVOS [19] and Equanox [8, 9]. These studies showed a mean rSO2 at 47%, 37%, and 42% in the ROSC group compared with 31%, 32% and 31% in the no ROSC group, and the differences between groups were statistically significant. Our study showed higher rSO2 in the ROSC group both with the INVOS™5100 and ROOT® O3. In these three previous studies, mechanical chest compressions were used in only two patients compared with all patients in our study. This could be one explanation for the higher rSO2 values in our study. A meta-analysis by Westfall et al. compared manual versus mechanical chest compressions in OHCA and concluded that mechanical compressions were superior regarding ROSC [22].

In our study, we found no statistically significant difference between ETCO2 in the ROSC and no ROSC groups. ETCO2 has a role during ALS to confirm the correct placement of an endotracheal tube. Prior studies have shown that ETCO2 < 1.3 kPa after 20 min of ALS had no survivors [23]. Rognås et al. found that four out of 22 patients received ROSC despite an ETCO2 < 1.3 kPa [24]. Kolar et al. found that median ETCO2 of 1.9 kPa during OHCA could distinguish between ROSC and no ROSC [25]. Prior studies have shown a relationship between a rise in ETCO2 and ROSC but no cut off value is agreed upon. ETCO2 is dependent on respiratory minute volume and can change when epinephrine or sodium bicarbonate is administered [26]. Heradstveit et al. showed that the cause of arrest, initial rhythm, bystander CPR, and time from arrest to ETCO2 measurement were confounding factors in interpreting the significance of ETCO2 values [27]. As in our study, Engel et al. found that rSO2 was superior to ETCO2 in predicting ROSC [28].

Feasibility of measuring cerebral oxygenation during cardiac arrest has been studied both in-hospital and in the pre-hospital setting, using different oximeters, with Equanox™ and INVOS™5100 being the most investigated [5–7, 10, 29]. Schewe et al. showed that rSO2 recordings were achieved in 89.9% of the intended recording time during OHCA [8]. Weatherall et al. showed that cerebral oximetry can be measured both during road and helicopter transports in healthy volunteers using Fore-Sight with a signal present > 70% of the time with 100% for road transported and 85.7% helicopter transported patients [30]. To our knowledge, no other study has assessed the feasibility of the ROOT® O3 during OHCA and transportation to the hospital.

To assess the usability of the two devices, we had one question in regard to interference with ALS for the RRC personnel to fill out after each study patient. The personnel rated the statement to a median of 2 (IQR 1–6) on a 10-point Numerical Rating Scale. From the answers with higher scores, we could read that there was problem with the battery running low, the device was heavy and difficult to carry along, the sensors fell off, or the application or documentation took focus away from the ALS. With nine people filling out the questionnaires, the interference rating could change after building up experience with the device. The place where the OHCA occurred and the situation could differ widely, meaning using the device could be easy in one case and more troublesome in another. Sensors falling off and connection problems between the sensor and cables can be a constant problem affecting the feasibility when applying sensors and transporting a patient from the place of arrest to the hospital. To be even more useful in a pre-hospital environment, the oximeter could be incorporated into the existing equipment or be small and easy to carry. Long battery capacity with a quick recharge are other advantageous features to avoid taking focus off of ALS. When measuring rSO2 with two different cerebral oximeters, we had expected a small difference in values but found a higher mean rSO2 in the ROOT O3® group with smaller confidence intervals than in the INVOS 5100™ group. We see potential in rSO2 guiding CPR, and it could contribute in part to making the decision of whether to continue resuscitation as well as if a patient could benefit from extracorporeal CPR.

Limitations.

The on-call physician decided whether to include a patient or not in the study, therefore, inclusion bias could have occurred. Since included patients ages ranged from 19 to 92, we believe even if there were an inclusion bias, it was minor. As we did not want our measurements to negatively impact ALS, sensor placements and the rSO2 measurements started at different times during the OHCA, at the place of arrest or during transportation. This means that we cannot compare all patients at the same timepoint, but as in prior studies, we present group-wise means for rSO2. rSO2 and ETCO2 were documented manually, leading to a risk of recall bias, but with the equipment used in the study, ETCO2 was routinely recorded manually by a physician who was familiar with this way of observation and documentation.

## Conclusion

Our results suggest that both the INVOS™5100 and ROOT® O3 can distinguish between ROSC and no ROSC during OHCA, and they can be used in a pre-hospital setting and during transport without affecting ALS.

## Electronic supplementary material

Below is the link to the electronic supplementary material.

Supplementary Material 1

## Data Availability

The datasets used and/or analyzed during the current study are available from the corresponding author on reasonable request.

## Abbreviations

OHCA: Out of hospital cardiac arrest
CPR: Cardiopulmonary resuscitation
rSO2: Regional cerebral oxygen saturation
ALS: Advanced life support
EMS: Emergency medical service
ETCO2: End tidal carbon dioxide
ROSC: Return of spontaneous circulation
