# Supplementary material for: Prehospital monitoring of cerebral circulation during out of hospital cardiac arrest ? A feasibility study
Source: Scand J Trauma Resusc Emerg Med. 2022 Dec 2;30:62. doi: 10.1186/s13049-022-01044-y (PMC9717485; doi:10.1186/s13049-022-01044-y)
Supplement: Supplementary file 1 — Supplementary Material 1 [file 13049_2022_1044_MOESM1_ESM.docx]

**Prehospital monitoring of cerebral circulation during out of hospital cardiac arrest – A feasibility study.**

**Anna Henningsson MD**. Department of Anesthesiology and Intensive Care Medicine, section for Cardiothoracic Anesthesia and Intensive Care, Sahlgrenska University Hospital, Gothenburg, Sweden. ***Corresponding author***

**Lukas Lannemyr MD PhD**. Department of Anesthesiology and Intensive Care Medicine, Sahlgrenska Academy, University of Gothenburg, Sahlgrenska University Hospital Gothenburg, Gothenburg, Sweden.

**Oskar Angerås MD PhD**. Department of Molecular and Clinical Medicine, Institute of Medicine, Sahlgrenska Academy, University of Gothenburg, Sweden, Region Västra Götaland, Sahlgrenska University Hospital, Department of Cardiology, Gothenburg, Sweden.

**Joakim Björås MD**. Department of Molecular and Clinical Medicine, Institute of Medicine, Sahlgrenska Academy, University of Gothenburg, Gothenburg, Sweden.

**Niklas Bergh, MD PhD**. Department of Molecular and clinical medicine, Institute of medicine Sahlgrenska academy, University of Gothenburg, Sweden & Department of cardiology, Sahlgrenska University hospital, Gothenburg, Sweden.

**Johan Herlitz MD PhD**. Centre for Prehospital Research; Faculty of Caring Science, Work Life and Social Welfare; University of Borås, Borås, Sweden.

**Bengt Redfors MD PhD**. Department of Anesthesiology and Intensive Care Medicine, Sahlgrenska Academy, University of Gothenburg, Section for Cardiothoracic Anesthesia and Intensive Care, Sahlgrenska University Hospital, Gothenburg, Sweden.

**Peter Lundgren MD PhD**. Department of Molecular and Clinical Medicine, Institute of Medicine, Sahlgrenska Academy, University of Gothenburg, Sweden, Prehospen - Centre for Prehospital Research, University of Borås, Sweden, Region Västra Götaland, Sahlgrenska University Hospital, Department of Cardiology, Gothenburg, Sweden.
